# Screening and Validation of Q-Markers for Daodi Authenticity of *Lycium barbarum* L. Using Multi-Component Quantification and Chemometrics

**DOI:** 10.3390/molecules31122059

**Published:** 2026-06-12

**Authors:** Yuying Hu, Kai He, Qun Luo, Ying Wang, Hongyu Jin, Feng Wei, Yongqiang Lin

**Affiliations:** 1 National Institutes for Food and Drug Control, Beijing 102629, China; huyuying@nifdc.org.cn (Y.H.); 3038729683@qq.com (Q.L.); wayi_1986@163.com (Y.W.); linyongqiang@nifdc.org.cn (Y.L.); 2 Ningxia Hui Autonomous Region Institute of Drug Control, Yinchuan 750002, China; woshihekai@163.com

**Keywords:** *Lycium barbarum* L., daodi authenticity, quality markers (Q-markers), chemometrics, polysaccharide, zeaxanthin dipalmitate, appearance traits, UPLC-MS/MS, AHP-CRITIC

## Abstract

To identify quality markers (Q-markers) for daodi authenticity evaluation of *Lycium barbarum* L., a comprehensive strategy integrating appearance trait analysis, multi-component quantification, and chemometrics was developed. Forty-five sample batches were collected from four major producing areas in China, namely Ningxia (NX), Gansu (GS), Qinghai (QH), and Inner Mongolia (NM). Appearance traits (50-fruit weight, moisture, and color) and the contents of polysaccharide, total sugar, betaine, zeaxanthin dipalmitate, and 27 small-molecule compounds, including flavonoids and phenolics, were determined using UV–vis spectrophotometry, HPLC-CAD, and UPLC-MS/MS. Pearson correlation analysis revealed a significant negative association between polysaccharide and total sugar (*r* = −0.344, *p* < 0.05), suggesting a possible allocation shift between the two carbohydrate fractions, while zeaxanthin dipalmitate strongly correlated with redness (*r* = 0.609, *p* < 0.01). Principal component analysis identified total sugar, polysaccharide, scopoletin, and scopolin as key discriminatory variables. AHP-CRITIC combined weighting highlighted polysaccharide (weight 0.195) and zeaxanthin dipalmitate (weight 0.157) as candidate core Q-markers. Top-ranked comprehensive scores predominantly belonged to samples from NX and GS, chemically supporting the traditional daodi authenticity. This dual-dimensional “efficacy–trait” framework provides a robust, traceable basis for origin authentication and quality standard improvement of *L. barbarum*.

## 1. Introduction

*Lycium barbarum* L. (Solanaceae), commonly known as goji berry, is a well-known medicinal and edible plant in China, with the dried ripe fruits (*Lycii Fructus*) officially listed in the Chinese Pharmacopoeia for their functions of tonifying the liver and kidneys and improving eyesight [1]. Historical textual research has indicated that the daodi region of *L. barbarum* has gradually shifted from Changshan, Tangyi, and Ganzhou (the Hexi Corridor) to NX and its adjacent areas, forming the traditional quality cognition characterized by “red color, moist texture, firmness, and few seeds” [2,3]. However, modern cultivation and breeding practices increasingly emphasize traits such as large fruit size, high sweetness, and high yield, potentially leading to a divergence between commercial appearance and medicinal quality [4,5]. This discrepancy has become a critical issue in the quality research and standard upgrading of *L. barbarum* fruits.

Recently, the concept of “quality markers” (Q-markers) was proposed by Liu et al. to address the limitations of current quality control systems for traditional Chinese medicines (TCMs) [6,7]. According to the Q-marker theory, ideal markers should be compounds that are correlated with efficacy, specific to the herb, measurable, traceable throughout the production process, and consistent with TCM theory [8,9]. Although previous studies have focused on the quantification of single components such as polysaccharide or betaine in *L. barbarum*, such approaches are insufficient to reflect the holistic quality of this multi-component herbal medicine [10]. In contrast, multi-component fingerprinting combined with chemometrics offers a powerful tool to reveal the overall chemical profile and to screen for potential Q-markers [11,12,13]. Recent studies have demonstrated the value of integrating chromatographic fingerprints, spectroscopic data, and multivariate statistical tools for the geographical discrimination and quality assessment of *L. barbarum* fruits [14,15,16].

Therefore, the present study aimed to establish a comprehensive quality evaluation method that integrates both traditional experience and modern scientific understanding. A total of 45 batches of samples were collected from four major medicinal *L. barbarum* fruit-producing regions (NX, GS, QH, and NM). The appearance traits (weight, moisture, color) and the contents of polysaccharide, total sugar, betaine, zeaxanthin dipalmitate, and 27 small-molecule compounds, including flavonoids and phenolics, were systematically determined. Various chemometric methods, including Pearson correlation analysis, ANOVA, PCA, cluster analysis, and AHP-CRITIC combined weighting, were used to investigate the chemical differences among regions and to screen for potential Q-markers [17,18].

Compared with previous studies that mainly focused on single-component quantification or chromatographic fingerprinting, the present work offers four distinct advances: (1) it is the first to integrate multi-component quantification with AHP-CRITIC combined weighting to construct a dual-dimensional “efficacy–trait” daodi authenticity evaluation system for *L. barbarum*; (2) it explicitly links the external quality trait (“bright red color”) with its chemical basis (zeaxanthin dipalmitate) and with pharmacological activities (vision protection); (3) it reports, for the first time, a negative correlation between polysaccharide and total sugar content in *L. barbarum* fruits, providing a chemical explanation for the traditional observation that sweeter and larger fruits are not necessarily medicinally superior; and (4) it establishes a validated LC-MS/MS method for 27 small-molecule compounds and reveals, for the first time, the regional distribution patterns of 19 detected secondary metabolites in *L. barbarum* fruits. This work provides a theoretical basis for quality control and standard improvement of *L. barbarum* fruits.

## 2. Results

### 2.1. External Traits of Samples

The external traits of the 45 batches showed clear differences among producing areas (Table 1). Samples from NM and QH generally exhibited higher fruit weight, indicating a larger commercial size. By contrast, samples from NX, GS, and QH tended to show higher red color values than those from NM. The color result is particularly meaningful because the red appearance of *L. barbarum* fruits is one of the best-known traditional quality cues. In the present dataset, the red color value was closely associated with zeaxanthin dipalmitate, which is the key carotenoid pigment in the fruit [19]. Therefore, the external trait of “bright red color” can be interpreted not only as a sensory feature but also as a chemically explainable quality signal.

### 2.2. Contents of Polysaccharide and Total Sugar

Polysaccharide and total sugar were successfully determined by the anthrone-sulfuric acid method (Table 2). The established calibration curve showed acceptable linearity, and the precision, repeatability, and recovery results supported the suitability of the method for routine quantification (Tables S2–S5, Figure S1). Among the four producing areas, QH samples showed higher total sugar accumulation, whereas the NX and GS samples generally exhibited relatively higher polysaccharide levels. This pattern suggests that sweetness-related sugar accumulation and efficacy-oriented polysaccharide accumulation may not increase synchronously, a phenomenon also observed in previous studies on *L. barbarum* fruits’ carbohydrate metabolism [20].

### 2.3. Contents of Betaine and Zeaxanthin Dipalmitate

Betaine and zeaxanthin dipalmitate are both important quality-related constituents in *L. barbarum* fruits (Table 3). Betaine is already an official quality control indicator in the Chinese Pharmacopoeia, whereas zeaxanthin dipalmitate is strongly associated with both fruit color and eye-health-related activity [21,22]. In the present study, zeaxanthin dipalmitate showed relatively high levels in NX samples and displayed a strong positive relationship with the red color value. This result supports its dual role as a traditional trait-related and efficacy-related marker. Betaine also showed a relatively favorable accumulation trend in some NX samples, suggesting that it can serve as a supportive rather than core marker in the geo-authenticity evaluation system.

### 2.4. Multi-Component Profiling of Secondary Metabolites

A multi-component LC-MS/MS method was established to determine 27 target compounds, among which 19 were detected in the investigated samples. These compounds mainly included coumarins, phenolic acids, and flavonoids. The complete quantitative data for the 19 detected compounds are provided in Table S12, and a regional summary (range and mean by producing region) is presented in Table 4. The method provided a broader chemical view of origin-dependent variation beyond the conventional official markers, and the linearity, precision, and recovery results supported its suitability (Tables S9–S11) [23]. Several compounds, such as scopoletin, scopolin, chlorogenic-acid-related derivatives, and flavonoid glycosides, exhibited coordinated variation patterns. This indicates that the chemical profile of *L. barbarum* fruits is shaped by biosynthetically related compound clusters rather than by isolated single markers, a pattern consistent with the phenolic profiling reported for *Lycium* species from different Chinese regions [24]. The non-detected compounds are gallic acid, caffeoylquinic acid, catechin, 1,3-O-dicaffeoylquinic acid, epicatechin, hesperidin, isorhamnetin-3-O-neohesperidoside, and morin. Possible reasons include (1) their concentrations in dried *L. barbarum* fruits may be below the limit of quantification (LOQ) of our method; (2) degradation during drying or storage; (3) ion suppression due to matrix effects in the UPLC-MS/MS system. The absence of these eight compounds does not preclude their presence at trace levels; future studies with improved sensitivity are needed to verify their occurrence and potential biological roles.

### 2.5. Chemometric Differentiation Among Producing Areas

#### 2.5.1. Pearson Correlation Analysis

**Figure 1:**
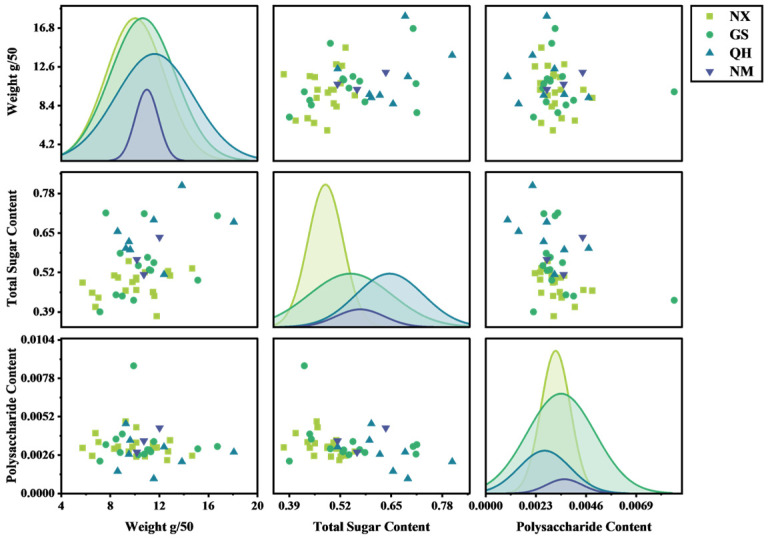
Scatter plot of the weight–polysaccharide–total sugar correlation matrix.

**Figure 2:**
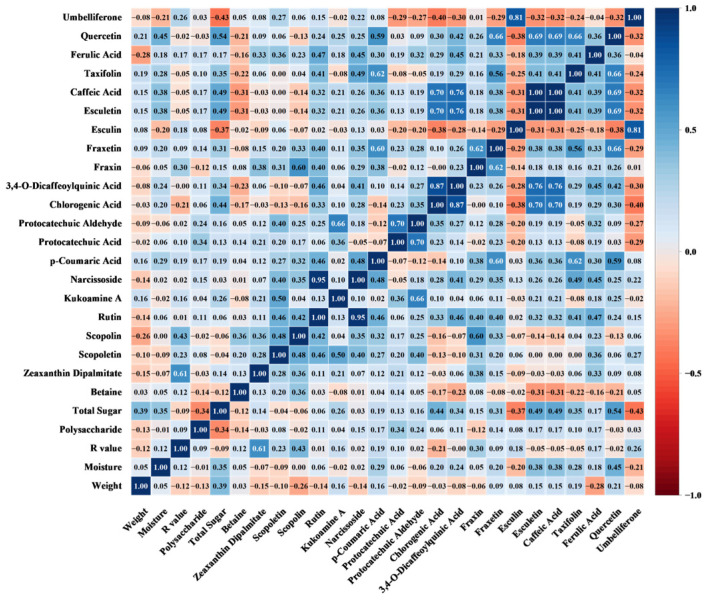
Correlation heat map.

Pearson correlation analysis, a fundamental statistical method for assessing linear relationships between variables [25], revealed significant synergistic and antagonistic relationships within the complex chemical constituent network of *L. barbarum* fruits (Table 5, Figure 1 and Figure 2). Weight was moderately positively correlated with total sugar content (*r* = 0.391, *p* < 0.01), suggesting that the accumulation of monosaccharides and oligosaccharides may be associated with increased fruit plumpness. In contrast, polysaccharide content was significantly negatively correlated with total sugar (*r* = −0.344, *p* < 0.05), suggesting a possible allocation shift between soluble sugar accumulation and functional polysaccharide synthesis in *L. barbarum*’s sugar metabolism.

Furthermore, fraxetin and caffeic acid (*r* = 1.000) showed an almost completely coordinated accumulation trend, suggesting a common biosynthetic origin (phenylpropanoids) and co-regulation under the same ecological conditions. Similarly, strong positive correlations were observed between rutin and narcissoside (*r* = 0.951) and between chlorogenic acid and 3,4-O-dicaffeoylquinic acid (*r* = 0.721). Notably, zeaxanthin dipalmitate content was significantly positively correlated with the R value (*r* = 0.609, *p* < 0.01), providing chemical evidence support for the traditional quality judgment the “the redder the fruit, the better the quality”.

#### 2.5.2. One-Way Analysis of Variance (ANOVA)

**Figure 3:**
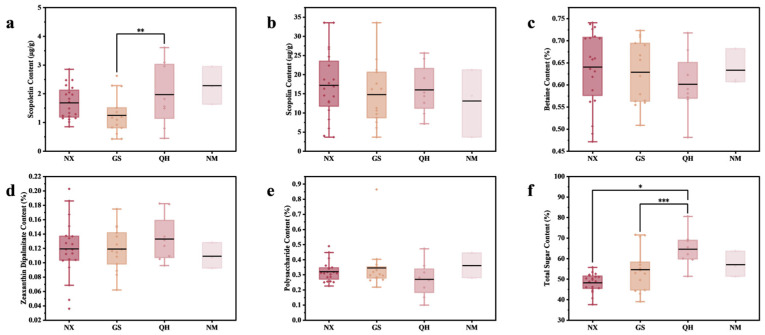
Box plot of six indices. (**a**) Scopoletin; (**b**) scopolin; (**c**) betaine; (**d**) zeaxanthin dipalmitate; (**e**) polysaccharide; (**f**) total sugar. *** indicates *p* < 0.001, ** indicates *p* < 0.005, * indicates *p* < 0.05.

One-way analysis of variance (ANOVA) is widely used in botanical and food chemistry studies to test for significant differences among group means [26]. In this study, ANOVA was performed on six indicators (scopoletin, scopolin, zeaxanthin dipalmitate, betaine, polysaccharide, and total sugar) across the 45 samples from different producing areas (Figure 3). The producing area was significantly associated with total sugar accumulation (*p* < 0.001); total sugar in QH was significantly higher than in GS (*p* < 0.001) and NX (*p* < 0.05). In contrast, polysaccharide content in NX and GS was higher than in QH, reinforcing the notion that polysaccharide and total sugar may exhibit a reciprocal pattern under different ecological conditions. Zeaxanthin dipalmitate levels were relatively high in NX, while variations in betaine and coumarin components among regions suggested that the geographic environment may exert selective effects on the accumulation of different metabolite classes.

#### 2.5.3. Principal Component Analysis (PCA)

**Figure 4:**
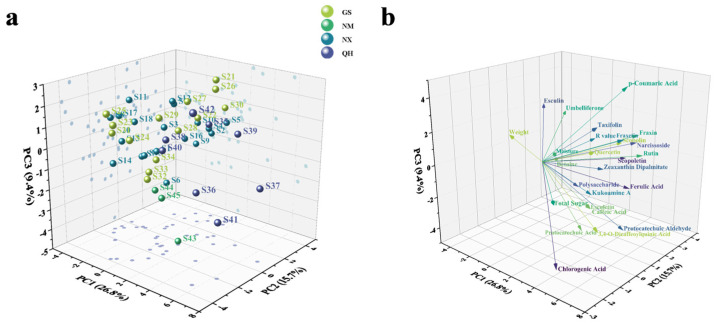
PCA (**a**) score plot and (**b**) loading plot.

Principal component analysis (PCA) is an unsupervised dimensionality reduction technique that has been extensively applied to evaluate differences in the quality of Chinese herbal medicines from various origins [27]. PCA of 26 indicators from 45 batches of samples revealed significant differences in the chemical characteristics of samples from different origins (Figure 4). The first two principal components (PC1 and PC2) together explained 44.95% of the variance. Total sugar content was a key negative indicator driving sample separation along the PC2 axis (loading: −0.467), whereas polysaccharide content (loading: 0.474) contributed positively, reflecting possible differences in primary metabolite allocation among producing areas.

Regarding secondary metabolites, scopoletin and scopolin were the core variables contributing positively to PC2 (loadings of 0.651 and 0.830, respectively). Their high loadings were associated with elevated scores for some NX samples, suggesting that NX may be a potential enrichment area for these bioactive coumarins. Additionally, zeaxanthin dipalmitate and betaine contributed positively to PC2 (loadings of 0.354 and 0.331, respectively), further enriching the multidimensional interpretation of PCA discrimination.

However, the low cumulative variance (44.95%) explained by PC1 and PC2 indicates that the full chemical complexity may not be captured by the first two components, which should be considered as a limitation when interpreting the PCA-based discrimination.

#### 2.5.4. Cluster Analysis

**Figure 5:**
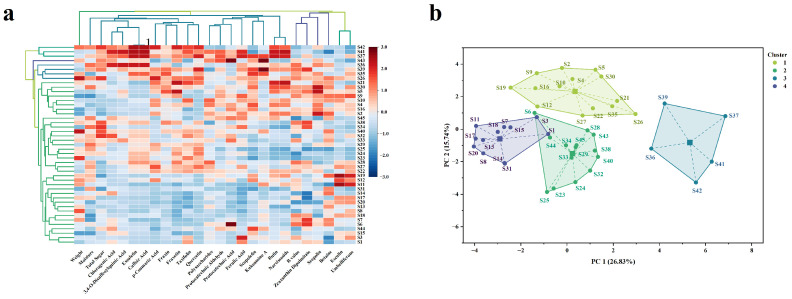
PCA (**a**) hierarchical clustering heatmap; (**b**) K-means clustering plot.

Hierarchical clustering analysis (HCA) and K-means clustering are complementary pattern recognition methods commonly used in the quality assessment of TCMs to visualize sample groupings [28,29]. In this study, both methods were used for cluster analysis (Figure 5). The hierarchical clustering heatmap (Figure 5a) visually displays the similarity and difference patterns across the 45 sample batches based on multi-index chemical constituents. Overall, the samples did not form independent branches strictly according to origin; instead, a structure of “cross-origin continuous gradient + local origin aggregation” was observed. Some NX and GS samples were highly interleaved in the heatmap, indicating strong chemical similarity, whereas QH samples, although not completely clustered into one class, were more concentrated in local regions and displayed a relatively consistent profile.

Taken together, the clustering results indicate that the chemical characteristics of the samples exhibit both geographic origin specificity and patterns of cross-origin chemical similarity. Hierarchical clustering emphasizes the continuity of chemical characteristics, showing a gradient transitional distribution, while K-means clustering highlights the concentration trend of QH samples. Both methods collectively demonstrate that geographic origin is an important, but not the sole, determinant of chemical characteristics; cultivation management, harvest maturity, and processing methods may also shape the final chemical profile.

### 2.6. Comprehensive Evaluation and Candidate Q-Markers

**Figure 6:**
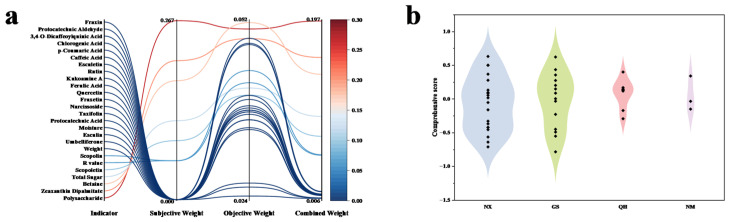
Comprehensive evaluation. (**a**) weight distribution; (**b**) score distribution of different origins.

To integrate expert knowledge and data-driven weighting, an AHP-CRITIC combined weighting model was established [30,31]. This strategy balanced pharmacological relevance, Q-marker logic, indicator variability, and inter-indicator conflict, thus improving the interpretability of the comprehensive quality evaluation (Table 6, Figure 6).

As shown in Table 6, the comprehensive scores of the 45 sample batches ranged from −0.785 to 0.629, and most of the top 10 ranked samples came from NX and GS. This result is highly consistent with the traditional understanding that Ningxia and its neighboring areas are the daodi producing areas of goji berry, providing further evidence supporting the rationality of daodi authenticity evaluation.

From the weight structure (Figure 6a), polysaccharide (weight 0.195) and zeaxanthin dipalmitate (weight 0.157) contributed the most to the comprehensive evaluation model, accounting together for over 35% and constituting the core dimension of quality evaluation. This outcome aligns closely with the Q-marker framework. Polysaccharides are the primary material basis for the immunomodulatory and antitumor effects of *L. barbarum* fruits [32,33,34], and their high weight reflects an emphasis on core efficacy. Zeaxanthin dipalmitate, as the key pigment component responsible for fruit redness, not only corresponds to the traditional empirical judgment of “good quality with red color” but also possesses well-documented antioxidant and visual protective activities [35,36,37], thereby linking traditional trait identification with modern pharmacological understanding.

Overall, this weight structure accurately covers the two dimensions of “efficacy and trait”: polysaccharide represents the intrinsic material basis of efficacy, while zeaxanthin dipalmitate represents the external color quality trait. The two complement each other and jointly constitute a quality evaluation framework that balances the inheritance of daodi authenticity experience with scientific understanding, in line with the core principles advocated by Q-marker theory, namely “efficacy-related, traditionally interpretable, measurable, and controllable.” Therefore, these two compounds are proposed as the most promising candidate Q-markers for geo-authentic *L. barbarum* fruits. Betaine may be considered as a supportive marker due to its moderate weight (0.139) and status as a pharmacopoeia indicator, although it alone cannot fully represent daodi authenticity. Scopolin and scopoletin, despite their lower weights (0.054 and 0.074, respectively), show high discriminatory power in PCA and are region-specific, thus serving as auxiliary markers for fine geographical differentiation.

Betaine may be considered as a supportive marker, while scopolin and scopoletin may serve as auxiliary characteristic markers for more refined regional discrimination.

## 3. Discussion

Daodi authenticity is the external manifestation formed by the comprehensive action of “origin, ecology, processing, and efficacy” in Chinese medicinal materials, and its scientific interpretation needs to return to a traceable and quantifiable material basis. Q-marker theory emphasizes that markers should simultaneously satisfy key attributes such as relevance to efficacy, uniqueness, measurability, traceability of quality, and consistency with TCM theory [6,8]. Based on this theoretical framework, this study systematically screened and validated the Q-markers of daodi authenticity for *L. barbarum* fruits.

*L. barbarum* polysaccharide, as the main material basis for the immunomodulatory and antitumor effects of *L. barbarum* fruits, is one of the quality control indicators in the 2025 edition of the Chinese Pharmacopoeia [1]. This study provides new data support for this indicator from the perspective of daodi authenticity: the polysaccharide contents of samples from NX and adjacent GS were significantly higher than those from QH, and the top-ranked samples in the comprehensive scores were highly concentrated in NX and GS, indicating an intrinsic consistency between the stable high accumulation of *L. barbarum* polysaccharide and the traditional daodi producing areas. It is noteworthy that total sugar was significantly negatively correlated with polysaccharide content, and total sugar drove the negative separation of samples in the PCA. This significant negative correlation is of particular interest from a pharmacological perspective. Modern research has established that *L*. *barbarum* polysaccharides are the primary active constituents responsible for the immunomodulatory, hepatoprotective, and renoprotective effects that underpin the traditional “tonifying liver and kidney” actions [32,33,34]. In contrast, while the sweet taste (gan wei) in TCM theory is associated with “tonifying” properties, the simple sugars (glucose, fructose, and sucrose) that dominate the total sugar fraction primarily contribute to commercial sweetness and palatability rather than the core therapeutic efficacy [38]. The daodi samples from NX and GS exhibited higher polysaccharide content together with lower total sugar. This chemical profile aligns with the traditional quality cognition that “sweeter and larger fruits are not necessarily medicinally superior” [20,32,39]. Thus, the observed negative correlation represents a coordinated chemical characteristic of authentic daodi *L. barbarum*.

Zeaxanthin dipalmitate content was extremely significantly positively correlated with fruit redness, providing a clear material basis for the traditional empirical judgment of “good quality with red color.” The content of this component was generally higher in NX, GS, and QH than in NM, showing stable inter-origin differences [15,16]. More importantly, as a carotenoid component, the pharmacological activity of zeaxanthin dipalmitate is highly consistent with the traditional eyesight-improving efficacy: modern studies have shown that carotenoids have blue-light filtering and retinal protective effects [22,35,36,37]. This means that the component simultaneously possesses the dual attributes of traditional trait characterization and modern efficacy relevance, making it a particularly representative Q-marker candidate.

In addition to the two candidate core markers above, betaine, as a pharmacopeial indicator, showed a trend of higher accumulation in NX samples and may serve as an auxiliary Q-marker to enhance the stability of the evaluation system [21]. Coumarin compounds such as scopolin and scopoletin contributed significantly to origin differentiation in the PCA and were associated with some NX samples, suggesting that they may serve as potential characteristic Q-markers for the fine identification of daodi producing areas [23,24]. However, the direct relationship of these two types of components with traditional efficacy still needs to be verified by spectrum-effect studies.

Compared with previously reported Q-marker studies for *L. barbarum*, which mainly focused on single bioactive compounds such as betaine or polysaccharide alone [10], the present work offers a dual-dimensional “efficacy–trait” framework. This framework aligns more closely with the five principles of Q-marker theory (effectiveness, specificity, measurability, transferability, and compatibility) [6,8]. Polysaccharide serves as the efficacy core based on its well-documented immunomodulatory, hepatoprotective, and renal protective activities, directly supporting the traditional “tonifying liver and kidney” actions [32,33,34]. Zeaxanthin dipalmitate bridges traditional traits (red color) with modern pharmacological understanding (antioxidant and vision protection) [35,36,37]. The auxiliary coumarins (scopolin and scopoletin) add regional specificity. This multi-layer, data-driven approach is more robust for quality standard upgrading than single-marker models.

At the methodological level, this study used correlation analysis, variance analysis, principal component analysis, cluster analysis, and the AHP-CRITIC combined weighting method to perform cross-validation among multiple methods, effectively avoiding the bias of a single statistical model [25,26,27,28,29,30,31]. The “continuous gradient + local aggregation” pattern presented by the clustering results is consistent with the understanding that daodi authenticity is a complex system jointly shaped by multiple factors: the chemical profile is constrained by geographic origin while also being regulated by ecological gradients and human intervention, showing the coexistence of discrete types and continuous variation [28,29]. The comprehensive scoring model based on AHP-CRITIC further integrates the two dimensions of efficacy and traits in a quantitative manner, making the evaluation’s conclusion more interpretable and operational.

This study still has certain limitations. First, the sampling was cross-sectional (only the 2023 harvest year), which may not capture inter-annual variability. Second, the number of NM samples was limited (*n* = 3), which may affect the comprehensive presentation of the chemical characteristics of this producing area. Third, the effects of different cultivated varieties on the chemical profiles have not yet been deeply analyzed, and cultivar differences could influence metabolite accumulation. Fourth, although all samples were processed under controlled drying and storage conditions, post-harvest handling might still affect certain chemical components. Fifth, the relationship between polysaccharides’ structural heterogeneity and activity remains to be explored [40,41,42]. Sixth, 8 of the 27 target compounds were not detected in any sample, which may be due to their low abundance in dried fruits, degradation during drying/storage, or matrix effects in the LC-MS/MS analysis, thus leading to an incomplete chemical fingerprint. Seventh, the relatively high recovery (112.04%) and moderate RSD (7.80%) of the polysaccharide assay may be attributed to matrix interference inherent to the anthrone–sulfuric acid method. Therefore, the reported polysaccharide values should be interpreted with caution, and complementary methods are recommended for confirmation. Eighth, the LOQ for each compound was estimated as the lowest calibration point, and matrix effects were not systematically evaluated. Although the good linearity (R^2^ > 0.99) and acceptable recoveries suggest that quantification was not severely compromised, the lack of formal LOQ determination and a dedicated matrix effect assessment is a methodological limitation. Finally, the proposed Q-markers (polysaccharide and zeaxanthin dipalmitate) are derived from chemometric correlations and model-based weighting; direct spectrum–effect relationship studies and external validation using an independent sample set have not been performed. Future studies may incorporate ecological factors such as light, altitude, and soil elements to construct an environment–metabolite coupling model; control the cultivar as a variable to separate geographical effects from the genetic background; and combine spectrum–effect correlation analysis to verify the synergistic contribution of the combination of polysaccharide and zeaxanthin dipalmitate to key efficacies such as “improving eyesight,” thereby closing the loop between efficacy and quality markers and facilitating the establishment of Q-marker standards [43,44,45,46,47].

## 4. Materials and Methods

### 4.1. Samples, Reagents, and Instruments

Samples: In total, 45 batches of *L. barbarum* fruits from different producing areas were collected and were jointly identified by the National Institutes for Food and Drug Control and the Ningxia Institute for Drug Control as the dried mature fruits of *L. barbarum* L. Samples S1–S20 were from NX, S21–S34 from GS, S35–S42 from QH, and S43–S45 from NM. Detailed information on the origin, cultivar identity, harvest date, drying conditions and storage conditions is provided in Table S1. Briefly, all 45 batches were harvested in autumn 2023. The main cultivars were Ningqi No. 1 and Ningqi No. 7. Drying was performed uniformly by hot air at 40 °C to a moisture content of ≤13%. Samples were stored in sealed containers at 25 °C protected from light. All samples were non-commercially sourced and were collected by our research team directly from the producing areas.

Reagents: Methanol and acetonitrile (HPLC grade) were purchased from Sigma (St. Louis, MO, USA); the water was ultrapure water. Zeaxanthin dipalmitate (Batch No. SGDH89, purity > 95%) was purchased from Shanghai Beiwanta Biotechnology Co., Ltd. (Shanghai, China). Anhydrous glucose, betaine, gallic acid, protocatechuic acid, protocatechuic aldehyde, aesculin A, catechin, kukoamine A, chlorogenic acid, scopolin, 1,3-O-dicaffeoylquinic acid, fraxetin, fraxin, caffeic acid, epicatechin, esculetin, 7-hydroxycoumarin, p-coumaric acid, scopoletin, taxifolin, ferulic acid, 3,4-O-dicaffeoylquinic acid, hesperidin, isorhamnetin-3-O-neohesperidoside, rutin, narcissin, and quercetin were all purchased from the National Institutes for Food and Drug Control (Beijing, China); caffeoylquinic acid, p-coumaric acid, taxifolin, and phellamurin reference substances were purchased from Shanghai Yuanye Bio-Technology Co., Ltd. (Shanghai, China).

Instruments: A UV-2700 UV–vis spectrophotometer (Shimadzu, Kyoto, Japan); an Ultimate 3000 HPLC system (Thermo Fisher, Waltham, MA, USA); Class-vp HPLC system (Shimadzu, Kyoto, Japan); an 8060 UHPLC-tandem mass spectrometry system (Shimadzu, Kyoto, Japan); the Milli-Q ultrapure water system (Millipore, Burlington, MA, USA).

### 4.2. Determination of External Traits

In total, 50 fruits from each sample were taken and weighed, and the weight of 50 fruits (g/50 fruits) was used as an indicator of the size of *L. barbarum* fruit samples. Moisture content was determined according to Method 2 “Drying method” of the moisture determination methods in Part IVof the 2025 edition of the Chinese Pharmacopoeia [1]. For color measurement, sample photographs were obtained under a consistent camera setup with identical illumination (D65 standard light source, 5500 K) and exposure conditions, and a gray card was used for white balance calibration. The photographs were then uploaded to the “QTCCOLOR” database (Qiantong Color Library, https://www.qtccolor.com, accessed on 15 October 2025), and the “image color recognition” mode was selected. For each sample, three positions (avoiding specular highlights and edges) were selected with the recognition area fully covered by the sample. The HEX color values (hexadecimal color code) returned by the tool were recorded and converted to RGB format. The final R, G, and B values for each sample were calculated as the average of the three measurements.

### 4.3. Determination of Polysaccharide and Total Sugar

An appropriate amount of an anhydrous glucose reference substance was accurately weighed and dissolved in water to prepare a solution containing 0.2 mg per mL. Aliquots of the reference solution (0.1, 0.2, 0.3, 0.4, 0.5, 0.8, and 1.0 mL) were precisely measured and transferred into separate 10 mL stoppered test tubes. The volume in each tube was adjusted to 1.0 mL with water. Then, 3 mL of an anthrone–sulfuric acid solution (prepared by dissolving 0.1 g of anthrone in 100 mL of sulfuric acid) was rapidly and accurately added. The mixture was immediately shaken, allowed to stand for 15 min, and then immediately cooled in an ice bath for another 15 min. After cooling, the absorbance was measured at 625 nm using a UV–vis spectrophotometer (Chinese Pharmacopoeia 2025, Part IV, General Chapter 0401) [1], with the solvent as a blank. The calibration curve was constructed by plotting absorbance against concentration.

For sample preparation, the sample was frozen and then quickly pulverized. Approximately 1 g of the powder was accurately weighed and placed in a 250 mL conical flask. Water (60 mL) was added, and the mixture was allowed to stand for 1 h, followed by shaking in a water bath at 80 °C for 2 h (120 r/min). The mixture was filtered while hot, and the filter and residue were washed with a small amount of hot water. The residue was transferred to a conical flask, and 25 mL of water was added. After further shaking for 1 h, the mixture was filtered while hot, and the filter and residue were again washed with hot water. The combined filtrates were cooled and diluted with water to a final volume of 100 mL. This solution was used as the test solution.

To obtain the polysaccharide assay solution, an aliquot of the test solution (2 mL) was precisely measured, mixed with 8 mL of anhydrous ethanol, and allowed to stand at 4 °C for 12 h. After centrifugation at 5000 r/min for 5 min, the supernatant was discarded. The precipitate was washed with 4 mL of 80% ethanol, followed by another round of centrifugation at 5000 r/min for 5 min. The supernatant was discarded, and the precipitate was dissolved in water and diluted to a final volume of 5 mL. For the total sugar assay solution, an aliquot of the test solution (0.5 mL) was precisely measured and diluted with water to 10 mL.

For determination, aliquots (1 mL) of both the polysaccharide assay solution and the total sugar assay solution were precisely measured. Following the same procedure as for the calibration curve (adding 3 mL of the anthrone–sulfuric acid solution, shaking, standing, cooling, and measuring the absorbance), the absorbance was measured. The content of anhydrous glucose in the test solution was calculated using the calibration curve.

### 4.4. Determination of Betaine

An amino-bonded silica gel column was used as the stationary phase, acetonitrile and water (85:15) were used as the mobile phase, and a CAD detector was used. The theoretical plate number calculated on the basis of the betaine peak should not be less than 3000. An appropriate amount of the betaine reference substance was accurately weighed and dissolved in water to prepare a solution containing 0.17 mg per 1 mL. For the test solution, about 1 g of sample powder was accurately weighed into a stoppered conical flask, 50 mL of methanol was accurately added, then the flask was stoppered and weighed, heated under reflux for 1 h, cooled, and reweighed. The lost weight was replenished with methanol, and the mixture was shaken well and filtered. An aliquot (2 mL) of the subsequent filtrate was pipetted onto a basic alumina solid-phase extraction column (2 g) and eluted with 30 mL of ethanol, and the eluate was collected and evaporated to dryness. The residue was dissolved in water, transferred to a 2 mL volumetric flask, diluted to volume with water, shaken well, and filtered. Determination was performed by HPLC (Chinese Pharmacopoeia 2025, Part IV, General Chapter 0512) [1], injecting 10 μL each of the reference solution and test solution.

### 4.5. Determination of Zeaxanthin Dipalmitate

The chromatographic column was an Agilent ZORBAX SB-C18 (250 × 4.6 mm). Methanol was used as Mobile Phase A and methanol-dichloromethane-n-hexane (50:30:20) as Mobile Phase B, and elution was carried out at A:B = 28:72, with a flow rate of 0.8 mL/min, a column temperature of 35 °C, and a detection wavelength of 454 nm. The theoretical plate number calculated on the basis of the zeaxanthin dipalmitate peak should not be less than 4000. An appropriate amount of the zeaxanthin dipalmitate reference substance was accurately weighed, dissolved, and diluted with an ethyl acetate-n-hexane (3:1) mixed solution to prepare a solution containing 0.1 mg per 1 mL. For the test solution, 2.5 g of sample powder was accurately weighed into a stoppered conical flask, and 5 mL of water was added and shaken to disperse. Then 15 mL of an ethyl acetate-n-hexane (3:1) mixed solution was added, shaken for 20 min, and centrifuged for 5 min (6000 r/min), and the supernatant was decanted. Another 10 mL of the mixed solution was added to the residue, shaken for 2 min, and centrifuged, and the supernatants were combined and diluted to 25 mL with the mixed solution. Determination was performed by HPLC (Chinese Pharmacopoeia 2025, Part IV, General Chapter 0512) [1], injecting 10 μL each of the reference solution and the test solution.

### 4.6. Multi-Component LC-MS/MS Analysis

A targeted LC-MS/MS method was established for 27 compounds using reversed-phase separation and multiple reaction monitoring (MRM) acquisition. Mixed standard solutions were prepared for calibration, and sample extracts were obtained with 70% methanol. The method was validated before quantitative application to the 45 batches. The 27 target compounds were selected on the basis of three criteria: (i) chemical representativeness (covering flavonoids, phenolic acids, coumarins, alkaloids, and carotenoids previously reported in *L. barbarum*); (ii) literature evidence of pharmacological relevance (e.g., immunomodulation, antioxidant, vision protection); and (iii) analytical feasibility (availability of reference standards and good response in MRM mode).

#### 4.6.1. Chromatographic Conditions

An octadecylsilane-bonded silica gel column (10 cm length, 2.1 mm inner diameter, 1.8 μm particle size) was used; a 0.1% formic acid solution (containing 5 mmol/L ammonium formate) was used as Mobile Phase A, with a methanol–0.1% formic acid solution (containing 5 mmol/L ammonium formate) (95:5) as Mobile Phase B. Gradient elution was performed according to Table S6, with a flow rate of 0.3 mL/min and a column temperature of 40 °C.

#### 4.6.2. Mass Spectrometry Conditions

Detection was performed using a triple quadrupole tandem mass spectrometer with an electrospray ionization (ESI) source. MRM mode was used. The reference retention times, ion scan modes, monitored ion pairs, and collision energies (CE) of each compound are shown in Table S7.

#### 4.6.3. Preparation of the Mixed Reference Solution

Accurately weigh an appropriate amount of each reference substance, dissolve in 70% methanol, and prepare a mixed standard solution containing 27 compounds. The specific concentrations are shown in Table S8.

#### 4.6.4. Preparation of the Test Solution

Accurately weigh about 3 g of the sample, accurately add 25 mL of a 70% methanol solution, shake at high speed for 10 min, centrifuge, filter the supernatant through a membrane, and collect the subsequent filtrate as the test solution.

#### 4.6.5. Determination Method

The 27 chemical constituents in *L. barbarum* fruits were simultaneously determined by UPLC-MS/MS. Accurately inject 1 μL each of the “mixed reference solution in 4.6.3” and the “test solution in 4.6.4” into the HPLC-tandem mass spectrometer for determination and calculation.

### 4.7. Chemometric and Statistical Analysis

Pearson correlation analysis, one-way ANOVA, PCA, hierarchical clustering analysis (HCA), K-means clustering, and AHP-CRITIC combined weighting were performed using Modelab 2024 (Chemmind Technologies Co., Ltd., Beijing, China), OriginPro 2025b (OriginLab Corporation, Northampton, MA, USA), R studio (2025.09.0 + 387), and related software packages. Differences were considered statistically significant at the predefined thresholds used in the original analyses.

Before performing one-way ANOVA, the normality of the data distribution was assessed using the Shapiro–Wilk test, and the homogeneity of variances was checked using Levene’s test. As shown in Tables S13 and S14, all variables met the normality assumption (*p* > 0.05) within each producing region, and Levene’s test confirmed the homogeneity of variances (*p* > 0.05) for all six variables. Therefore, parametric ANOVA was considered appropriate.

The AHP-CRITIC combined weighting method was used to determine the weights of evaluation indicators. Subjective weights were determined using the AHP method. Five experts in TCM quality research and analytical chemistry participated in the pairwise comparisons. Based on knowledge of the pharmacologically active material basis and Q-markers, the relative importance relationships among evaluation indicators were determined. All 27 evaluation indicators were divided into 8 priority groups, and the importance of each group was assigned according to the 1–9 scale method to construct an 8-order judgment matrix (Table S15). In this matrix, 1 indicates equal importance, 3 is slightly important, 5 is obviously important, 7 is strongly important, and 9 is extremely important, while 2, 4, 6, and 8 are intermediate values between the abovementioned judgments. The consistency ratio (CR) was calculated as 0.078 (<0.10), indicating acceptable consistency.

Objective weights were determined using the CRITIC method. The CRITIC method comprehensively determines the objective weights on the basis of the contrast intensity of the evaluation indicators and the conflict among indicators and has the advantage of simultaneously considering both the magnitude of indicator variability and the correlations among indicators. First, the standard deviation of each indicator after Z-score standardization was calculated to reflect contrast intensity; second, the correlation coefficient matrix among the indicators was calculated, and the cumulative degree of conflicting indicators 1 − *r_jk_* was used to measure conflict among indicators; multiplying the standard deviation by the conflict yielded the amount of information contained in each indicator, which was then normalized to obtain the objective weight of each indicator.

Combined weights were determined using a linear weighted combination method. To ensure that the evaluation results could reflect both the leading role of professional judgment and the statistical characteristics of the data, proportions were set for subjective weights (*α* = 0.7) and objective weights (1 − *α* = 0.3). The combined weights of each indicator were calculated using the formula *w_combined_* = *α* × *w_subjective_* + (1 − *α*) × *w_objective_* and normalized again to obtain the final weight vector. To test robustness, a sensitivity analysis was performed by varying α from 0.5 to 0.9 in steps of 0.1. The top two Q-markers (polysaccharide and zeaxanthin dipalmitate) remained unchanged across this range (Table S16). This combined approach effectively balances professional experience and data information, providing a scientific basis for the comprehensive quality evaluation of *L. barbarum* fruits [30,31].

## 5. Conclusions

In summary, based on Q-marker theory and through multi-index determination and chemometric analysis, this study clarified that polysaccharide and zeaxanthin dipalmitate can be considered as candidate core Q-markers for evaluating the daodi authenticity of *L. barbarum* fruits. Polysaccharide represents the intrinsic material basis of efficacy, whereas zeaxanthin dipalmitate links traditional traits with modern pharmacology. Together, they constitute a dual-dimensional evaluation framework of “efficacy–trait.” The significant negative correlation between polysaccharide and total sugar suggests a possible resource allocation shift between soluble sugar and functional polysaccharide in *L. barbarum* sugar metabolism, providing an explanation for why fruits that are sweeter and larger are not necessarily medicinally superior. This study provides a scientific basis for improving the quality standards and an origin traceability system of *L. barbarum* fruits.

## Figures and Tables

**Table 1:** Weight, moisture and color measurement results.

No.	Weight g/50	Moisture %	HEX	R Value	G Value	B Value
S1	8.307	8	#953D34	149	61	52
S2	6.777	8.04	#A63B2D	166	59	45
S3	9.807	8.16	#A84635	168	70	53
S4	10.121	7.1	#94372F	148	55	47
S5	8.632	7.44	#AA372A	170	55	42
S6	10.116	8.56	#B53829	181	56	41
S7	12.641	6.56	#A53B2D	165	59	45
S8	14.678	7.38	#793029	121	48	41
S9	6.52	6.36	#AF4B37	175	75	55
S10	9.242	7.64	#99352C	153	53	44
S11	12.705	8.04	#9F362C	159	54	44
S12	12.868	9.05	#A1382A	161	56	42
S13	11.477	7.63	#902B23	144	43	35
S14	9.5	8.35	#752C22	117	44	34
S15	5.73	6.52	#83382D	131	56	45
S16	7.032	7.71	#A3372D	163	55	45
S17	11.591	8.97	#9D3728	157	55	40
S18	10.827	6.43	#B05C39	176	92	57
S19	11.788	9.34	#8E332C	142	51	44
S20	10.123	6.81	#6F3329	111	51	41
S21	11.3	8.52	#961E1A	150	30	26
S22	8.465	8.95	#A93228	169	50	40
S23	15.139	7.86	#772720	119	39	32
S24	11.043	8.91	#6C1A17	108	26	23
S25	11.177	9.65	#8B372D	139	55	45
S26	16.74	9.69	#B04131	176	65	49
S27	8.976	9.63	#A02F26	160	47	38
S28	9.896	9.3	#B64C3F	182	76	63
S29	8.803	10.29	#922D1F	146	45	31
S30	10.287	10.04	#AA3B2D	170	59	45
S31	7.153	9.66	#9C2E22	156	46	34
S32	10.767	9.26	#77261D	119	38	29
S33	11.549	9.26	#AE422D	174	66	45
S34	7.635	10.29	#9F4234	159	66	52
S35	12.373	6.89	#AA4D39	170	77	57
S36	9.515	12.83	#BB4336	187	67	54
S37	9.617	9.09	#81322E	129	50	46
S38	11.538	8.79	#B3463B	179	70	59
S39	8.587	9.44	#B7463D	183	70	61
S40	18.08	8.63	#9C3E32	156	62	50
S41	12.017	8.23	#9E4A3F	158	74	63
S42	10.164	7.35	#794541	121	69	65
S43	10.737	9.85	#5D1E19	93	30	25
S44	9.264	7.93	#862825	134	40	37
S45	13.842	9.9	#7B2D29	123	45	41

R, G, and B represent the red, green, and blue colour channel values in the RGB colour space. Unit: % (*w*/*w*, mass percentage).

**Table 2:** Results of polysaccharide and total sugar content determination.

No.	Polysaccharide Content (%)	Total Sugar Content (%)	No.	Polysaccharide Content (%)	Total Sugar Content (%)
S1	0.3030	51.05	S24	0.2963	56.97
S2	0.4086	40.65	S25	0.2951	52.92
S3	0.3170	48.87	S26	0.3181	70.62
S4	0.4480	46.20	S27	0.4029	44.23
S5	0.3168	50.34	S28	0.8642	42.84
S6	0.3439	50.29	S29	0.2774	58.29
S7	0.2258	51.78	S30	0.2622	54.24
S8	0.2555	53.36	S31	0.2189	39.03
S9	0.2548	45.32	S32	0.2668	71.32
S10	0.4887	45.99	S33	0.3518	55.23
S11	0.2849	52.49	S34	0.3304	71.57
S12	0.3598	50.98	S35	0.3153	51.34
S13	0.3396	45.52	S36	0.2652	62.10
S14	0.2837	55.73	S37	0.3599	59.48
S15	0.3082	48.69	S38	0.1000	69.27
S16	0.3479	43.68	S39	0.1503	65.49
S17	0.3093	44.26	S40	0.2799	68.55
S18	0.2506	52.09	S41	0.4441	63.62
S19	0.3117	37.62	S42	0.2801	56.29
S20	0.2495	49.64	S43	0.3571	51.31
S21	0.2821	52.66	S44	0.4723	59.94
S22	0.3680	44.63	S45	0.2147	80.57
S23	0.3028	49.47			

Unit: % (*w*/*w*, mass percentage).

**Table 3:** Results of betaine determination.

No.	Betaine Content (%)	Zeaxanthin Dipalmitate Content (%)	No.	Betaine Content (%)	Zeaxanthin Dipalmitate Content (%)
S1	0.507	0.137	S24	0.555	0.098
S2	0.741	0.134	S25	0.509	0.062
S3	0.472	0.118	S26	0.723	0.142
S4	0.588	0.113	S27	0.620	0.136
S5	0.658	0.167	S28	0.563	0.150
S6	0.710	0.186	S29	0.657	0.089
S7	0.706	0.203	S30	0.714	0.151
S8	0.631	0.112	S31	0.709	0.108
S9	0.731	0.137	S32	0.667	0.118
S10	0.705	0.126	S33	0.694	0.116
S11	0.489	0.105	S34	0.579	0.175
S12	0.618	0.103	S35	0.591	0.132
S13	0.705	0.118	S36	0.679	0.123
S14	0.727	0.036	S37	0.581	0.136
S15	0.564	0.104	S38	0.567	0.181
S16	0.660	0.127	S39	0.623	0.182
S17	0.562	0.048	S40	0.718	0.109
S18	0.636	0.151	S41	0.607	0.107
S19	0.738	0.093	S42	0.611	0.128
S20	0.664	0.069	S43	0.682	0.092
S21	0.690	0.114	S44	0.481	0.105
S22	0.560	0.125	S45	0.572	0.096
S23	0.565	0.083			

Unit: % (*w*/*w*, mass percentage).

**Table 4:** Regional distribution of secondary metabolites.

Compound	Region	Range	Mean	Compound	Region	Range	Mean
Scopoletin	NX	0.4287–3.0952	1.612	Fraxin	NX	0.0212–0.4613	0.146
	GS	0.4287–2.6232	1.370		GS	0.0237–0.3453	0.156
	QH	0.4510–3.6106	1.672		QH	0.0425–0.4085	0.194
	NM	1.6361–2.9506	2.282		NM	0.1825–0.2401	0.213
Scopolin	NX	3.523–42.104	17.08	Fraxetin	NX	0.0135–0.0659	0.034
	GS	7.582–37.399	16.72		GS	0.0164–0.0492	0.034
	QH	7.183–25.640	15.01		QH	0.0198–0.0485	0.035
	NM	3.585–21.257	13.10		NM	0.0320–0.0608	0.045
Rutin	NX	15.470–75.381	36.05	Esculin	NX	0.2764–7.3093	1.910
	GS	19.448–71.724	36.42		GS	0.4604–5.1785	1.716
	QH	25.686–75.381	40.05		QH	0.2764–1.0520	0.744
	NM	49.691–74.963	63.40		NM	0.5300–0.7011	0.626
Kukoamine A	NX	14.930–79.559	37.79	Esculetin	NX	0.2381–1.8231	0.728
	GS	24.350–76.669	44.03		GS	0.2381–1.4429	0.800
	QH	29.204–132.865	59.58		QH	0.5939–1.8231	1.044
	NM	27.154–48.354	37.95		NM	0.7456–2.1856	1.261
Narcissoside	NX	0.9029–4.0814	2.198	Caffeic Acid	NX	0.4761–3.6462	1.470
	GS	1.3317–3.6465	2.167		GS	0.4761–2.8858	1.471
	QH	1.4285–4.0814	2.297		QH	0.8150–3.6462	2.099
	NM	2.2653–4.2965	3.042		NM	1.4912–4.3713	2.523
p-Coumaric Acid	NX	4.6633–20.8018	10.13	Taxifolin	NX	0.0505–0.2061	0.113
	GS	4.6284–14.4122	9.826		GS	0.0668–0.2516	0.132
	QH	3.1244–12.4258	8.795		QH	0.0620–0.1705	0.112
	NM	5.4579–16.7853	10.47		NM	0.0761–0.1961	0.126
Protocatechuic Acid	NX	0.2080–0.6923	0.409	Ferulic Acid	NX	6.551–24.352	14.69
	GS	0.2486–0.5108	0.394		GS	6.551–19.363	11.52
	QH	0.3117–1.1861	0.528		QH	11.987–24.352	15.70
	NM	0.3731–0.6628	0.528		NM	13.881–21.931	18.02
Protocatechuic Aldehyde	NX	0.1364–0.5164	0.268	Quercetin	NX	0.0063–0.2115	0.059
	GS	0.1795–0.3374	0.256		GS	0.0546–0.1461	0.091
	QH	0.2513–1.0207	0.405		QH	0.0282–0.2115	0.093
	NM	0.1430–0.4818	0.336		NM	0.0258–0.1217	0.072
Chlorogenic Acid	NX	3.967–93.013	21.77	Umbelliferone	NX	0.0458–0.1683	0.091
	GS	4.855–52.805	18.96		GS	0.04996–0.1139	0.083
	QH	19.325–52.805	28.80		QH	0.0144–0.0992	0.072
	NM	28.386–93.013	52.58		NM	0.0458–0.06697	0.060
3,4-O-Dicaffeoylquinic Acid	NX	0.0149–0.7440	0.142				
	GS	0.0269–0.1958	0.101				
	QH	0.0888–0.7440	0.274				
	NM	0.2311–1.4827	0.702				

In total, 19 components were detected, while the remaining 8 components were not detected. Unit: μg/g.

**Table 5:** Results of pearson correlation analysis.

Correlation Coefficient (*r*)	Polysaccharide Content	Weight	Total Sugar Content
Polysaccharide Content	1	−0.125	−0.344 *
Weight	−0.125	1	0.391 **
Total Sugar Content	−0.344 *	0.391 **	1

* indicates a significant correlation at the 0.05 level (bilateral), and ** indicates a significant correlation at the 0.01 level (bilateral).

**Table 6:** Comprehensive evaluation results.

Rank	No.	Origin	Score	Rank	No.	Origin	Score	Rank	No.	Origin	Score
1	S10	NX	0.628523	16	S41	QH	0.136371	31	S38	QH	−0.172235
2	S26	GS	0.620369	17	S16	NX	0.130483	32	S29	GS	−0.229711
3	S6	NX	0.499524	18	S40	QH	0.128044	33	S42	QH	−0.294443
4	S2	NX	0.497138	19	S35	QH	0.116018	34	S1	NX	−0.329982
5	S28	GS	0.433101	20	S9	NX	0.095453	35	S3	NX	−0.369673
6	S37	QH	0.39792	21	S21	GS	0.08501	36	S8	NX	−0.420986
7	S5	NX	0.366532	22	S7	NX	0.082826	37	S15	NX	−0.424222
8	S30	GS	0.354169	23	S12	NX	0.052872	38	S24	GS	−0.450536
9	S43	NM	0.339069	24	S19	NX	0.015716	39	S14	NX	−0.456429
10	S4	NX	0.275823	25	S22	GS	0.002534	40	S31	GS	−0.491653
11	S27	GS	0.273037	26	S32	GS	−0.034608	41	S23	GS	−0.552592
12	S34	GS	0.200797	27	S45	NM	−0.036019	42	S11	NX	−0.565467
13	S36	QH	0.166899	28	S13	NX	−0.053914	43	S20	NX	−0.641047
14	S33	GS	0.146947	29	S44	NM	−0.151614	44	S17	NX	−0.711731
15	S39	QH	0.143853	30	S18	NX	−0.162417	45	S25	GS	−0.785049

## Data Availability

The original contributions presented in this study are included in the article and/or Supplementary Materials. Further inquiries can be directed to the corresponding authors.

## Supplementary Materials

The following supporting information can be downloaded at https://www.mdpi.com/article/10.3390/molecules31122059/s1. Table S1: Information of 45 batches of *L. barbarum* samples. Figure S1: Standard curve. Table S2: Linearity test results. Table S3: Precision test results. Table S4: Reproducibility test results. Table S5: Recovery test results. Table S6: Gradient of the mobile phase. Table S7: Mass spectrometric conditions for 27 chemical constituents. Table S8: Concentrations of 27 compounds in the mixed standard solution. Table S9: Linearity test results. Table S10: Precision test results. Table S11: Recovery test results. Table S12: Determination results of multiple components. Table S13: Normality (Shapiro–Wilk) test results for each variable by producing region. Table S14. Homogeneity of variance (Levene) test for all variables. Table S15. The 8 × 8 judgment matrix used for AHP priority groups. Table S16. Sensitivity analysis of combined weighting.
