# Supplementary material for: Screening and Validation of Q-Markers for Daodi Authenticity of Lycium barbarum L. Using Multi-Component Quantification and Chemometrics
Source: Molecules. 2026 Jun 12;31(12):2059. doi: 10.3390/molecules31122059 (PMC13304736; doi:10.3390/molecules31122059)
Supplement: Supplementary file 1 [file molecules-31-02059-s001.zip › Revised Supplementary Information-molecules-4342430.pdf]

Supplementary Information-Revised

Screening and Validation of Q-Markers for Daodi Authenticity of Lycium barbarum L. Using Multi-Component Quantification and Chemometrics

1. Samples

Table S1. Information of 45 Batches of *L. barbarum* Samples

No.	Region	Origin (Specific to Village/Town)	Cultivar Identity	Harvest Date
S1	NX	Liuyao Village, Sanhe Town, Haiyuan County, Ningxia	Ningqi No.1	July 6, 2023
S2	NX	Liuyao Village, Sanhe Town, Haiyuan County, Ningxia	Ningqi No.7	July 13, 2023
S3	NX	Siying Village, Sanhe Town, Haiyuan County, Ningxia	Ningqi No.7	July 6, 2023
S4	NX	Laocheng, Xi'an Town, Haiyuan County, Ningxia	Ningqi No.7	July 3, 2023
S5	NX	Huangwan, Maying Village, Jiatang Township, Haiyuan County, Ningxia	Ningqi No.7	July 23, 2023
S6	NX	Hongguan Village, Hongsibu District, Ningxia	Nongkeyuan No.608	June 28, 2023
S7	NX	Dahe Village, Dahe Township, Hongsibu District, Ningxia	Ningqi No.10	June 20, 2023
S8	NX	Lujiayingzi Village, Yanzidun Township, Huinong District, Ningxia	Ningqi No.7	June 24, 2023
S9	NX	Dongyonggu 8th Team, Miaotai Township, Huinong District, Ningxia	Ningqi No.1	July 2, 2023
S10	NX	Juhuatai Base, Juhuatai Village, Hexi Town, Tongxin County, Ningxia	Ningqi No.7	July 8, 2023
S11	NX	Dakong'erzhuang, Xiamaguan Town, Tongxin County, Ningxia	Ningqi No.7	August 12, 2023
S12	NX	Tongjiquan Village, Huamachi Town, Yanchi County, Ningxia	Ningqi No.7	June 26, 2023
S13	NX	Institute of Wolfberry Research, Garden Farm, Xixia District, Yinchuan City, Ningxia	Mixed variety	July 17, 2023
S14	NX	Tuanjie Village, Zhenbeibao Town, Xixia District, Yinchuan City, Ningxia	Ningqi No.7	June 17, 2023
S15	NX	Hongmaonanguo Wolfberry Tourist Garden, Xixia District, Ningxia	Mixed variety	June 29, 2023
S16	NX	Zhengqihong Wolfberry Industry Development Company Base, Ningxia	Ningqi No.7	July 18, 2023
S17	NX	Dikengzi MingSha Base, MingSha Town, Zhongning County, Ningxia	Ningqi No.10	June 28, 2023
S18	NX	Qixin Zhou Tower Base, Kangtan Village, ZhouTa Township, Zhongning County, Ningxia	Ningqi No.7	July 2, 2023
S19	NX	Antan Pian, Enhe Township, Zhongning County, Ningxia	Ningqi No.10	July 19, 2023
S20	NX	Dazhanchang Village, Dazhanchang Town, Zhongning County, Ningxia	Ningqi No.10	July 24, 2023
S21	GS	Yitiaoshan Farm, Jingtai County, Gansu	Ningqi No.1	July 18, 2023
S22	GS	Changcheng Village, Caowotou Town, Jingtai County, Baiyin, Gansu	Ningqi No.7	July 15, 2023
S23	GS	Xiaohonggou Village, Beitan Town, Jingyuan County, Baiyin City, Gansu	Ningqi No.7	July 10, 2023
S24	GS	Shahe Township, Guazhou County, Jiuquan City, Gansu	Ningqi No.7	July 26, 2023
S25	GS	Fuquan Village, Shuangta Township, Guazhou County, Jiuquan City, Gansu	Ningqi No.7	August 2, 2023
S26	GS	Xigou Third Team, Jinta Town, Jinta County, Jiuquan City, Gansu	Ningqi No.7	August 6, 2023
S27	GS	Shangjitaizi Village, Huangzhaowan Town, Yumen City, Jiuquan City, Gansu	Ningqi No.1	July 15, 2023
S28	GS	Liangzigou Village, Huangzhaowan Town, Yumen City, Jiuquan City, Gansu	Ningqi No.7	July 16, 2023
S29	GS	Xiadonghao Village, Xiaxihao Town, Yumen City, Jiuquan City, Gansu	Ningqi No.7	August 12, 2023
S30	GS	Liangzigou Village, Huangzhaowan Town, Yumen City, Jiuquan City, Gansu	Ningqi No.7	August 1, 2023
S31	GS	Fangjiadunzi, Peijia Ying Town, Gulang County, Wuwei City, Gansu	Ningqi No.7	August 12, 2023
S32	GS	Zhonghegou Village, Xiqu Town, Minqin County, Wuwei City, Gansu	Ningqi No.7	August 1, 2023
S33	GS	Daba Village, Xiqu Town, Minqin County, Wuwei City, Gansu	Ningqi No.9	July 5, 2023
S34	GS	Xintan Village, Caiqi Town, Minqin County, Wuwei City, Gansu	Ningqi No.1	June 25, 2023
S35	QH	Lubei Village, Zongjia Town, Dulan County, Qinghai	Ningqi No.7	August 17, 2023

S36	QH	Heishabao, Zongjia Town, Dulan County, Qinghai	Ningqi No.1	August 20, 2023
S37	QH	Qinghai Wanlihong Green Ecological Development Co., Ltd.	Ningqi No.7	August 20, 2023
S38	QH	Hexi Farm 12th Company, Guo Lemude Town, Golmud, Qinghai	Ningqi No.7	August 22, 2023
S39	QH	Dagole Township, Golmud, Qinghai	Ningqi No.1	August 25, 2023
S40	QH	Hexi Farm 3rd Company, Guo Lemude Town, Golmud, Qinghai	Ningqi No.7	August 22, 2023
S41	QH	Qinghai Yilin Wolfberry Technology Development Co., Ltd.	Ningqi No.1	June 25, 2023
S42	QH	Qinghai Yilin Wolfberry Technology Development Co., Ltd.	Ningqi No.1	June 25, 2023
S43	NM	Bayantaohai Farm, Dengkou County, Bayannur City, Inner Mongolia	Ningqi No.7	July 15, 2023
S44	NM	Qianjin Village, Shahai Town, Bayannur City, Inner Mongolia	Cunqi	July 18, 2023
S45	NM	Fenshui Village, Xianfeng Town, Bayannur City, Inner Mongolia	Ningqi No.7	July 10, 2023

*All 45 batches were dried under the same conditions (hot-air drying to ≤ 13 % moisture), stored in sealed containers at 25 °C protected from light, and were commercially sourced (randomly collected from local purchasing points/markets).

2. Methodological Investigation Results on the Determination of Polysaccharide and Total Sugar Content

2.1. Linearity

Aliquots (0.1, 0.2, 0.3, 0.4, 0.5, and 0.8 mL) of the reference solution (0.2015 mg/mL) were precisely measured and transferred to separate 10 mL stoppered test tubes. The volume in each tube was adjusted to 1.0 mL with water. Then, 3 mL of anthrone-sulfuric acid solution was rapidly and accurately added. The mixture was immediately shaken, allowed to stand for 15 min, and then immediately cooled in an ice bath for another 15 min. After cooling, the absorbance was measured at 625 nm, with the solvent as a blank. The calibration curve was plotted as absorbance vs. concentration, yielding the equation $y = 8.1537x - 0.002$ ($r^2 = 0.9953$), indicating good linearity (Table S2, Figure S1).

2.2. Precision

The same reference solution was measured six consecutive times. The relative standard deviation (RSD) of the absorbance values was 0.35%, demonstrating good precision (Table S3).

2.3. Reproducibility

Five portions of the same sample (1 g each) were accurately weighed and analyzed using the same method. The average polysaccharide content was found to be 0.484% with an RSD of 5.36% (Table S4).

2.4. Recovery

A sample with known polysaccharide content (1 g) was accurately weighed and processed according to the extraction method. After alcohol precipitation, the precipitate was dissolved in water and diluted to 5 mL. An aliquot (0.5 mL) of this solution was mixed with 0.05 mL of the glucose reference solution (0.2015 mg/mL), and the volume was adjusted to 0.5 mL with water. The mixture was analyzed following the same method, and the recovery was calculated. The average recovery was 112.04% with an RSD of 7.80% (Table S5).

Table S2. Linearity Test Results

n	Volume of Reference Solution (mL)	Concentration (mg/mL)	Absorbance
1	0.1	0.0202	0.149
2	0.2	0.0403	0.308
3	0.3	0.0602	0.484
4	0.4	0.0806	0.687
5	0.5	0.1008	0.857
6	0.8	0.1612	1.282

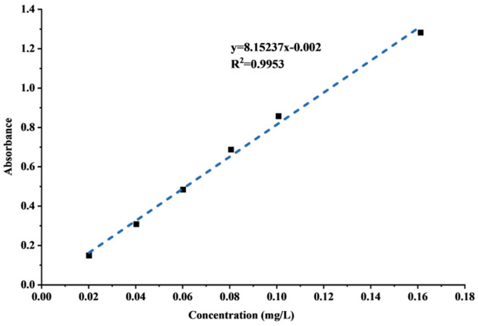

Figure S1. Standard Curve

Table S3. Precision Test Results

n	Absorbance
1	0.294
2	0.295
3	0.295
4	0.296
5	0.296
6	0.297
Average	0.296
RSD (%)	0.35

Table S4. Reproducibility Test Results

n	Polysaccharide Cntent (%)
1	0.515
2	0.489
3	0.467
4	0.493
5	0.458
Average	0.484
RSD (%)	5.36

Table S5. Recovery Test Results

n	Sample Weight (g)	Amount in Sample (μg)	Amount Added (μg)	Total Detected (μg)	Recovery (%)	Average Recovery (%)	RSD (%)
1	1.202	11.63536	10.075	24.382	126.51		
2	1.144	11.07392	10.075	22.568	114.09		
3	1.090	10.55120	10.075	21.359	107.27	112.04	7.80
4	1.482	14.34576	10.075	24.986	105.61		
5	1.387	13.42616	10.075	24.180	106.74		

3. Multi-component LC-MS/MS Analysis Methodological Investigation and Determination Results

3.1. Chromatographic Conditions

Table S6. Gradient of the Mobile Phase

Time/min	Mobile Phase A/%	Mobile Phase B/%
0-1	70	30
1-12	70→0	30→100
12-14	0	100

3.2. Mass Spectrometry Conditions

Table S7. Mass Spectrometric Conditions for 27 Chemical Constituents

No.	Name	RT	Polarity	Quantitative Ion Pair			Qualitative Ion Pair		
				Precursor Ion	Product Ion	CE1	Precursor Ion	Product Ion	CE2
1	Gallic acid	2.99	-	168.9	79	25	168.9	125.1	16
2	Caffeoylquinic acid	3.91	-	353	191	25	353	85	48
3	Protocatechuic acid	4.12	-	153.1	109.1	15	153.1	91.0	26
4	Protocatechuic aldehyde	4.69	-	137	108	26	137	92	28
5	Esculin	5.00	+	341	179	22	341	133	45
6	Catechin	5.12	-	289.2	109.1	25	289.2	203.1	19
7	Kukoamine A	5.19	+	531.3	293.1	30	531.3	222.1	35
8	Chlorogenic acid	5.20	-	353.2	191.1	20	353.2	85.0	42
9	Scopolin	5.23	+	355.1	192.9	10	355.1	133	35
10	1,3-O-Dicaffeoylquinic acid	5.32	-	515.1	353.1	20	515.1	191.0	32
11	Esculetin	5.52	+	179	123	20	179	133	20
12	Fraxin	5.53	-	369.1	207	20	369.1	192	23
13	Caffeic acid	5.63	-	179.0	135.1	18	179.0	106.9	23
14	Epicatechin	5.78	-	289.2	109.1	25	289.2	203.1	19
15	Fraxetin	5.95	+	209	194	22	209	166	25
16	Umbelliferone	6.22	+	163	107	22	163	77	35
17	p-Coumaric acid	6.31	-	163	119	15	163	91	22
18	Scopoletin	6.39	-	190.9	176.2	17	190.9	120	25
19	Taxifolin	6.45	-	303	285	8	303	125	18
20	Ferulic acid	6.48	-	193.1	134.1	18	193.1	178.0	15
21	3,4-O-Dicaffeoylquinic acid	6.63	-	515.1	353.1	20	515.1	173.0	30
22	Hesperidin	6.95	-	609.2	301.2	28	609.2	164.2	57
23	Isorhamnetin-3-O-neohesperidoside	7.01	-	623	314	28	623	299	42
24	Rutin	7.23	+	611.2	303.1	16	611.2	465.1	16
25	Morin	7.69	+	303.1	153.2	35	303.1	229.1	35
26	Narcissoside	7.73	-	623	299	40	623	315	6
27	Quercetin	8.18	-	300.9	151	22	300.9	178.9	18

3.3. Preparation of Mixed Reference Solution

Table S8. Concentrations of 27 Compounds in the Mixed Standard Solution

No.	Name	Concentration (mg/mL)	No.	Name	Concentration (mg/mL)
1	Gallic acid	1.075	15	Fraxetin	2.000
2	Caffeoylquinic acid	1.000	16	Umbelliferone	2.000

3	Protocatechuic acid	1.000	17	p-Coumaric acid	4.000
4	Protocatechuic aldehyde	1.000	18	Scopoletin	0.940
5	Esculin	2.000	19	Taxifolin	1.000
6	Catechin	2.000	20	Ferulic acid	1.000
7	Kukoamine A	0.460	21	3,4-O-Dicaffeoylquinic acid	2.000
8	Chlorogenic acid	1.000	22	Hesperidin	1.000
9	Scopolin	0.460	23	Isorhamnetin-3-O-neohesperidoside	1.000
10	1,3-O-Dicaffeoylquinic acid	2.000	24	Rutin	1.000
11	Esculetin	2.000	25	Morin	1.000
12	Fraxin	0.560	26	Narcissoside	1.000
13	Caffeic acid	1.000	27	Quercetin	2.000
14	Epicatechin	1.000			

3.4. Linearity

According to the concentration ranges of the analytes in samples, reference solutions of different concentrations of each compound were prepared and injected for determination. Calibration curves were plotted using concentration versus peak area. The results showed that all compounds had good linear relationships within the proposed injection ranges, with correlation coefficients r^2 greater than 0.99 (Table S9).

Table S9. Linearity Test Results

No.	Name	Linear Equation	r^2	Linear range (ng/mL)
1	Gallic acid	y=329.44x+6353.	0.9959	2.1-2100
2	Caffeoylquinic acid	y=302.33x+3304.9	0.9993	2-2000
3	Protocatechuic acid	y=541.3x-2781.1	0.9996	2-1000
4	Protocatechuic aldehyde	y=823.99x+17672	0.9991	4-4000
5	Esculin	y=13019x+341404	0.9933	4-1000
6	Catechin	y=28.601x+707.9	0.9951	4-2000
7	Kukoamine A	y=10406x-1E+07	0.9964	192-48111
8	Chlorogenic acid	y=448.99x+66206	0.9943	20-5000
9	Scopolin	y=2443.1x+337971	0.9953	92.7-23175
10	1,3-O-Dicaffeoylquinic acid	y=1771x+5805.5	0.9985	4-400
11	Esculetin	y=2982.3x+61136	0.9967	4-2000
12	Fraxin	y=386.55x-2619	0.9999	4.5-4500
13	Caffeic acid	y=2665.7x+157360	0.9946	4-4000
14	Epicatechin	y=38.825x-734.82	0.9999	20-20000
15	Fraxetin	y=24194x+629156	0.9965	2-2000
16	Umbelliferone	y=60631x+983172	0.9946	2-1000
17	p-Coumaric acid	y=3947x+704199	0.9988	20-20000
18	Scopoletin	y=1717.4x-39015	0.9999	18.7-18700
19	Taxifolin	y=339.48x+2813.5	0.9993	2-2000
20	Ferulic acid	y=126.77x-15000	0.9994	20-20000
21	3,4-O-Dicaffeoylquinic acid	y=1154.4x+11141	0.9995	4-400
22	Hesperidin	y=324.4x-48.05	0.9995	2-2000
23	Isorhamnetin-3-O-neohesperidoside	y=341.94x-1241	0.9999	2-2000

24	Rutin	y=374.84x+409818	0.9983	200-200000
25	Morin	y=878.03x+1470.8	0.9994	2-200
26	Narcissoside	y=456.52x+2097.8	0.9999	4-4000
27	Quercetin	y=3871.4x-5437.2	0.9986	4-400

3.5. Precision

The same reference solution was injected continuously six times, and injection precision was calculated from peak areas. The results showed that the RSD of injection precision for all compounds met the requirements (Table S10).

Table S10. Precision Test Results

No.	Name	1	2	3	4	5	6	Mean Peak Area	RSD
1	Gallic acid	12139	12405	13301	13608	12743	12231	12738	4.71
2	Caffeoylquinic acid	10111	9709	10685	9965	9876	9479	9971	4.13
3	Protocatechuic acid	16633	16380	17206	16028	15281	15840	16228	4.12
4	Protocatechuic aldehyde	53048	62238	66080	60843	59788	60139	60356	7.04
5	Esculin	1223608	1246384	1445527	1357488	1255003	1238761	1294462	6.80
6	Catechin	2275	1872	2160	2022	2105	1947	2064	7.11
7	Kukoamine A	16952943	17325719	16159261	14571132	14302794	14082720	15565762	9.16
8	Chlorogenic acid	234330	220404	219666	213409	211164	198005	216163	5.56
9	Scopolin	3544352	3558860	3966519	3629428	3603075	3570373	3645435	4.40
10	1,3-O-Dicaffeoylquinic acid	116548	119713	125874	116323	114925	109362	117124	4.67
11	Esculetin	221907	211641	215693	208187	217849	214268	214924	2.23
12	Fraxin	24356	24100	24792	24318	24707	24223	24416	1.12
13	Caffeic acid	221907	211641	232603	208187	217849	214268	217743	4.00
14	Epicatechin	12385	11387	11823	12082	11711	10894	11714	4.48
15	Fraxetin	1181567	1096435	1094252	1074661	1052594	1054032	1092257	4.36
16	Umbelliferone	2641995	2616641	2838399	2690787	2680164	2758050	2704339	3.01
17	p-Coumaric acid	1681416	1675215	1783609	1687160	1678188	1684811	1698400	2.47
18	Scopoletin	477116	465218	476258	477029	445562	451985	465528	2.98
19	Taxifolin	12303	11450	11931	12937	12874	11206	12117	5.94
20	Ferulic acid	32996	30122	32146	29674	31072	30352	31060	4.13
21	3,4-O-Dicaffeoylquinic acid	108434	106857	96251	89917	89644	83324	95738	10.55
22	Hesperidin	11212	12819	12888	11985	11572	12353	12138	5.56
23	Isorhamnetin-3-O-neohesperidoside	9725	9631	9840	9126	8927	9180	9405	3.98
24	Rutin	1251760	1166772	1266512	1158125	1174405	1167154	1197455	4.03
25	Morin	30457	34263	32133	35643	35999	34740	33873	6.37
26	Narcissoside	28273	26377	28059	25473	25579	25436	26533	4.95
27	Quercetin	311913	297835	297099	281546	264844	262611	285975	6.90

3.6. Recovery

Accurately weigh 1.5 g of the same sample, successively add 1 mL of mixed standard solution and 24 mL of 70% methanol solution, prepare and determine according to the method under "Preparation of test solution" in 4.6.4, and calculate the spiked recovery. The results showed that the recoveries of all compounds ranged from 63.6%

to 123.5%, meeting the determination requirements (Table S11).

Table S11. Recovery Test Results

No.	Name	1	2	3	4	5	6	Average Recovery	RSD
1	Gallic acid	107.80	119.19	107.96	103.89	102.31	96.04	106.20	7.27
2	Caffeoylquinic acid	65.79	68.58	68.05	64.51	66.11	63.85	66.15	2.84
3	Protocatechuic acid	133.12	139.38	116.35	107.83	125.16	119.17	123.50	9.32
4	Protocatechuic aldehyde	126.25	125.21	122.61	115.09	125.46	123.61	123.04	3.34
5	Esculin	72.17	77.51	71.88	67.63	63.05	60.47	68.78	9.21
6	Catechin	78.98	96.74	80.28	94.44	89.00	93.37	88.80	8.50
7	Kukoamine A	95.37	122.24	101.31	118.67	122.20	111.20	111.83	10.15
8	Chlorogenic acid	77.26	83.77	64.84	69.85	59.51	80.35	72.60	13.01
9	Scopolin	103.20	100.90	97.59	94.87	91.21	92.62	96.73	4.87
10	1,3-O-Dicaffeoylquinic acid	61.64	64.86	65.57	60.66	64.58	64.23	63.59	3.09
11	Esculetin	120.46	121.53	112.05	109.43	122.37	126.59	118.74	5.55
12	Fraxin	157.43	157.91	148.71	146.09	143.92	143.98	149.67	4.30
13	Caffeic acid	90.43	82.99	86.96	89.26	86.88	90.03	87.76	3.17
14	Epicatechin	116.08	120.00	110.44	114.21	114.64	113.97	114.89	2.72
15	Fraxetin	73.47	74.52	72.72	69.25	68.15	69.50	71.27	3.69
16	Umbelliferone	87.54	87.88	87.52	84.87	80.56	78.99	84.56	4.60
17	p-Coumaric acid	96.77	104.29	103.67	110.22	98.68	107.58	103.53	4.94
18	Scopoletin	84.68	100.09	83.40	90.70	87.49	85.28	88.61	6.98
19	Taxifolin	101.48	102.65	102.27	99.25	89.83	85.45	96.82	7.58
20	Ferulic acid	94.16	104.28	120.56	108.82	114.82	107.18	108.30	8.37
21	3,4-O-Dicaffeoylquinic acid	82.08	80.16	82.83	79.71	77.78	69.70	78.71	6.05
22	Hesperidin	103.99	100.64	103.01	99.23	90.92	103.20	100.17	4.86
23	Isorhamnetin-3-O-neohesperidoside	90.62	96.18	90.27	89.14	85.74	84.70	89.44	4.57
24	Rutin	117.81	112.73	122.08	118.38	114.76	126.18	118.66	4.12
25	Morin	94.82	93.48	94.24	97.85	91.85	103.15	95.90	4.24
26	Narcissoside	110.15	104.29	118.34	104.55	100.09	92.15	104.93	8.46
27	Quercetin	92.26	98.36	90.69	85.80	80.94	81.08	88.19	7.77

3.7. Determination Results

Table S12. Determination Results of Multi Components

No.	Scopoletin	Scopolin	Rutin	Kukoamine A	Narcissoside	p-Coumaric Acid	Protocatechuic Acid	Protocatechuic Aldehyde	Chlorogenic Acid	3,4-O-Dicaffeoylquinic Acid	Fraxin	Fraxetin	Esculin	Esculetin	Caffeic Acid	Taxifolin	Ferulic Acid	Quercetin	Umbelliferone
S1	1.2174	18.745	40.911	39.185	2.64811	5.6522	0.2786	0.24774	20.952	0.13336	0.06330	0.02330	0.6428	0.4743	0.9487	0.16198	18.418	0.01350	0.06799
S2	1.8275	35.166	57.599	46.235	3.32171	11.8379	0.4753	0.33018	5.398	0.03093	0.18117	0.03148	1.1168	0.5237	1.0474	0.09499	18.413	0.01404	0.09132
S3	1.7148	14.313	34.938	43.617	2.28183	5.8212	0.2844	0.23462	9.826	0.05854	0.09338	0.02774	1.7790	0.4823	0.9645	0.15220	22.743	0.01185	0.11328
S4	2.8557	26.668	58.072	41.556	3.24550	12.0276	0.6013	0.34657	6.345	0.05106	0.20346	0.03953	1.8250	0.5918	1.1835	0.14298	13.990	0.01461	0.09894
S5	1.9711	42.104	60.709	33.519	2.95929	10.3308	0.4275	0.37562	11.062	0.30744	1.09628	0.06586	1.8011	0.5120	1.0240	0.09856	11.210	0.01171	0.09504
S6	1.4872	18.725	31.758	39.069	1.56055	5.4999	0.9850	0.30157	20.021	0.11524	0.12551	0.02244	1.4707	0.5560	1.1119	0.08123	10.918	0.01006	0.07994
S7	1.1040	17.788	31.045	42.658	1.76572	4.6633	0.4309	0.23806	20.296	0.14887	0.09083	0.01936	1.3077	0.3030	0.6059	0.07331	10.417	0.01341	0.08100

S8	1.1505	8.288	28.138	34.718	1.65206	7.3751	0.2724	0.17033	5.144	0.01489	0.04998	0.01606	1.3407	0.3714	0.7427	0.10500	8.423	0.00996	0.10265
S9	1.9799	24.684	33.413	26.907	2.13845	6.1959	0.5060	0.40297	12.667	0.20480	0.24244	0.04471	3.3230	0.4222	0.8443	0.07935	19.694	0.01293	0.13254
S10	2.2069	22.379	47.803	36.196	3.12558	12.1293	0.4331	0.33700	3.967	0.03002	0.10121	0.02306	2.8783	0.6864	1.3728	0.19309	17.240	0.01002	0.08869
S11	0.8559	5.955	38.740	62.525	2.83927	5.1883	0.2498	0.15368	6.407	0.02430	0.02122	0.01349	7.3093	0.3305	0.6610	0.08550	8.681	0.00956	0.16832
S12	2.4804	16.897	41.888	41.718	2.02913	12.9251	0.4664	0.23873	6.794	0.04514	0.14068	0.03323	3.9549	0.7456	1.4913	0.11099	9.876	0.00700	0.11818
S13	1.2353	12.763	18.785	43.177	1.08862	6.0713	0.2080	0.21760	7.140	0.08759	0.07836	0.01957	1.4137	0.4389	0.8778	0.05049	9.998	0.00881	0.08114
S14	1.2897	16.322	33.105	21.439	2.00723	5.1880	0.2398	0.25236	30.304	0.07985	0.03331	0.02019	0.6209	0.5386	1.0771	0.06260	7.480	0.01201	0.06112
S15	2.0474	13.026	35.703	31.123	2.49527	5.6800	0.2830	0.22900	21.361	0.22642	0.11262	0.01631	1.8929	0.5229	1.0459	0.06796	11.617	0.00627	0.09687
S16	2.4752	27.209	45.353	27.983	2.28778	8.4355	0.6365	0.19730	8.870	0.07891	0.18262	0.03581	1.9092	0.5633	1.1266	0.06400	15.726	0.00731	0.09760
S17	1.3283	10.726	30.927	24.326	1.68313	5.1309	0.3097	0.20945	4.000	0.03288	0.09286	0.01981	2.9451	0.4530	0.9061	0.05460	8.582	0.00748	0.11353
S18	1.1540	3.523	31.898	45.667	1.95333	8.2140	0.3236	0.15489	8.820	0.04184	0.04698	0.01715	2.7098	0.6766	1.3533	0.09457	9.501	0.02056	0.10656
S19	2.2962	14.564	50.657	39.247	3.13698	7.7993	0.3808	0.31127	6.625	0.06139	0.08612	0.02592	5.1785	0.4268	0.8536	0.10382	13.037	0.00773	0.14861
S20	1.0175	3.993	35.993	42.731	2.43913	4.6284	0.2486	0.21769	9.873	0.02689	0.02366	0.01635	2.6428	0.5047	1.0095	0.06678	6.551	0.01209	0.11387
S21	2.2671	24.028	71.724	27.876	4.35307	13.4495	0.3160	0.25979	4.855	0.11899	0.28322	0.06524	0.5476	0.2381	0.4761	0.25156	14.475	0.13250	0.05360
S22	1.0956	20.638	51.156	44.936	2.86365	12.0020	0.4537	0.33084	8.031	0.15065	0.23027	0.04170	1.0967	0.8230	1.6461	0.16239	14.101	0.09146	0.10730
S23	0.4287	7.582	21.140	24.350	1.46707	7.4028	0.3672	0.17951	13.676	0.15459	0.16786	0.03842	0.8805	1.0385	2.0771	0.18122	10.005	0.09148	0.05549
S24	0.8134	9.701	21.360	24.436	1.33168	8.9156	0.4255	0.20639	15.648	0.15881	0.16374	0.04732	0.6066	1.0369	2.0737	0.13098	13.949	0.13073	0.05666
S25	0.6090	3.144	15.470	26.608	0.90290	10.7009	0.3491	0.13638	6.239	0.12087	0.09388	0.04184	0.4439	0.8667	1.7333	0.13806	8.887	0.12065	0.04996
S26	2.2844	21.246	39.594	48.262	2.89409	20.8018	0.4998	0.26686	8.609	0.17726	0.28309	0.04920	2.5550	1.0058	2.0116	0.17715	16.781	0.14607	0.09456
S27	1.3629	16.296	29.430	45.222	1.86305	14.4122	0.4155	0.25187	7.002	0.12349	0.19992	0.04223	2.8141	0.8598	1.7197	0.15596	16.944	0.13780	0.10630
S28	1.5153	6.087	32.391	37.467	2.33702	10.9056	0.4639	0.22656	18.646	0.19575	0.10668	0.04997	1.9365	1.2017	2.4035	0.13988	12.284	0.09639	0.10047
S29	1.1933	17.665	26.357	36.484	1.79925	11.9563	0.4336	0.24135	8.006	0.09829	0.21293	0.03700	1.2865	0.8471	1.6942	0.15040	13.947	0.10136	0.06896
S30	2.6232	37.399	63.684	31.714	3.64646	11.3215	0.4503	0.32790	14.442	0.12635	0.34527	0.03751	0.9826	0.5057	1.0114	0.14590	13.340	0.06838	0.11385
S31	0.4271	10.640	19.448	14.930	1.39653	5.5478	0.2841	0.15874	14.899	0.10976	0.10499	0.02697	0.5422	0.5507	1.1015	0.11588	7.632	0.05461	0.05287
S32	0.9156	11.276	33.048	37.727	1.99682	6.8205	0.5725	0.33737	44.054	0.17063	0.07288	0.03600	0.8000	0.9659	1.9319	0.17541	10.767	0.09210	0.09624
S33	0.8234	8.703	30.598	23.715	1.83181	6.8765	0.6923	0.20433	24.216	0.41346	0.10933	0.02965	0.4929	0.8819	1.7637	0.11879	14.152	0.07512	0.07234
S34	1.1680	16.202	20.244	27.496	1.15859	6.7958	0.5108	0.27029	7.346	0.13767	0.17262	0.02822	0.4604	0.5344	1.0687	0.13203	19.363	0.05964	0.05688
S35	3.0952	24.151	36.426	76.669	1.94128	11.3850	0.3216	0.27572	10.659	0.12628	0.46127	0.03498	0.9770	1.4429	2.8858	0.11278	14.789	0.10951	0.10475
S36	1.8216	25.640	49.277	46.797	2.62144	7.5400	0.3117	0.25125	52.805	0.74404	0.24460	0.01975	0.8150	1.7596	3.5191	0.10715	16.454	0.06598	0.07665
S37	3.6106	9.806	75.381	79.559	4.08136	12.4258	0.5583	0.51644	36.720	0.54383	0.26566	0.04849	1.0520	1.8231	3.6462	0.17045	24.352	0.21147	0.09924
S38	1.4869	15.309	26.151	29.204	1.43879	6.4630	0.3141	0.27001	19.927	0.19731	0.36876	0.04377	0.9690	1.0507	2.1014	0.11246	14.243	0.09032	0.06337
S39	2.9583	14.389	54.239	107.601	3.08649	12.1546	0.3215	0.34521	19.325	0.36772	0.40848	0.04479	0.5893	0.9533	1.9066	0.14694	17.504	0.18238	0.09599
S40	1.5578	12.630	31.984	59.872	1.84428	7.1933	0.4071	0.30479	23.675	0.20301	0.26262	0.03929	0.6931	0.9345	1.8690	0.15469	13.620	0.09837	0.06329
S41	0.4510	7.183	25.686	132.865	1.42846	3.1244	1.1861	1.02071	30.904	0.18441	0.04250	0.04531	0.2764	0.9075	1.8150	0.06201	12.138	0.05670	0.01437
S42	0.7968	19.077	34.232	56.332	2.00498	3.8700	0.4866	0.36672	31.342	0.08882	0.06175	0.02606	0.3381	0.5939	1.1877	0.12392	11.987	0.02818	0.06314
S43	2.2581	21.257	49.691	48.354	2.26526	5.4579	0.6628	0.38435	28.386	0.23110	0.18253	0.03196	0.5300	0.7456	1.4912	0.07614	18.242	0.02576	0.06697
S44	2.9506	14.458	74.963	27.154	4.29652	9.1787	0.5475	0.48182	93.013	1.48266	0.21691	0.04230	0.6476	2.1856	4.3713	0.19609	21.931	0.12171	0.04741
S45	1.6361	3.585	65.554	38.326	3.56529	16.7853	0.3731	0.14300	36.356	0.39452	0.24009	0.06078	0.7011	1.8527	3.7053	0.20609	13.881	0.16913	0.04576

*A total of 19 components were detected, while the remaining 8 components were not detected. Unit: μg/g.

4. ANOVA Assumption Tests

To verify the assumptions of one-way ANOVA, normality was assessed using the Shapiro-Wilk test, and homogeneity of variances was assessed using Levene's test. The results are presented in Table S13 and S14.

Table S13. Normality (Shapiro-Wilk) Test Results for Each Variable by Producing Region

**Variable**	**Producing region**	**Shapiro-Wilk W**	**df**	**Sig. (*p*)**

Polysaccharide	NX	0.950	20	0.360
	GS	0.922	14	0.237
	QH	0.952	8	0.736
	NM	0.983	3	0.747
Total sugar	NX	0.969	20	0.726
	GS	0.991	14	1.000
	QH	0.875	8	0.170
	NM	0.990	3	0.813
Betaine	NX	0.909	20	0.060
	GS	0.902	14	0.119
	QH	0.953	8	0.745
	NM	0.790	3	0.091
Zeaxanthin dipalmitate	NX	0.950	20	0.360
	GS	0.922	14	0.237
	QH	0.952	8	0.736
	NM	0.983	3	0.747
Scopoletin	NX	0.936	20	0.204
	GS	0.900	14	0.114
	QH	0.937	8	0.584
	NM	0.999	3	0.941
Scopolin	NX	0.950	20	0.360
	GS	0.922	14	0.237
	QH	0.952	8	0.736
	NM	0.983	3	0.747

*For NM (n=3), the Shapiro-Wilk test has limited power, but the p-values are all > 0.05, indicating no severe violation of normality.

Table S14. Homogeneity of Variances (Levene) Test for All Variables

**Variable**	**Levene Statistic**	**df1**	**df2**	**Sig. (*p*)**
Polysaccharide	0.311	3	41	0.817
Total sugar	0.552	3	41	0.650
Betaine	0.931	3	41	0.434
Zeaxanthin dipalmitate	0.311	3	41	0.817
Scopoletin	2.819	3	41	0.051
Scopolin	0.311	3	41	0.817

5. AHP-CRITIC Weighting Details

The 27 evaluation indicators were divided into 8 priority groups (G1-G8) based on chemical and pharmacological relevance. The groups are: G1: Polysaccharide, G2: Zeaxanthin dipalmitate, G3: Betaine, G4: Total sugar, G5: Scopoletin, G6: Scopolin, G7: R value (color), G8: Other indicators (remaining 20 compounds).

Pairwise comparisons were scored using the 1-9 scale (1 indicates equal importance, 3 slightly important, 5 obviously important, 7 strongly important, and 9 extremely important, while 2, 4, 6, and 8 are intermediate values). The full judgment matrix is shown below. Consistency ratio (CR) = 0.078 < 0.10, indicating acceptable consistency.

Table S15. 8×8 Judgment Matrix for AHP Priority Groups

	**G1**	**G2**	**G3**	**G4**	**G5**	**G6**	**G7**	**G8**
G1	1	1	2	2	3	5	5	9

G2	1	1	1	2	2	3	3	7
G3	1/2	1	1	2	2	3	3	6
G4	1/2	1/2	1/2	1	1	2	2	4
G5	1/3	1/2	1/2	1	1	2	2	3
G6	1/5	1/3	1/3	1/2	1/2	1	1	2
G7	1/5	1/3	1/3	1/2	1/2	1	1	2
G8	1/9	1/7	1/6	1/4	1/3	1/2	1/2	1

*The matrix is reciprocal ($a_{ji} = 1/a_{ij}$). CR = 0.078 < 0.10, confirming acceptable consistency.

To evaluate the robustness of the AHP-CRITIC combined weighting system, the subjective weight coefficient α was varied from 0.5 to 0.9 in steps of 0.1. For each α, the combined weights of polysaccharide and zeaxanthin dipalmitate were recalculated, and their ranks among all 27 indicators were determined. The top two Q-markers (polysaccharide and zeaxanthin dipalmitate) remain unchanged across the entire range of α, confirming the robustness of the weighting system. The detailed results are presented below.

Table S16. Sensitivity Analysis of Combined Weighting

α	Polysaccharide Weight	Zeaxanthin Dipalmitate Weight	Polysaccharide Rank	Zeaxanthin Dipalmitate Rank
0.5	0.1576	0.1274	1	2
0.6	0.1790	0.1431	1	2
0.7	0.1951	0.1567	1	2
0.8	0.2219	0.1745	1	2
0.9	0.2433	0.1902	1	2
